# The Role of Age, Political Affiliation, and Framing in Attitudes Toward Hispanic and Latinx Immigrants

**DOI:** 10.1093/geroni/igaa057.326

**Published:** 2020-12-16

**Authors:** Mengzhao Yan, Zachary Gassoumis, Kathleen Wilber, Sheila Salinas Navarro

**Affiliations:** University of Southern California, Los Angeles, California, United States

## Abstract

The United States is experiencing rapid aging and increasing racial and ethnic diversity. Nevertheless, political rhetoric about immigrants has stoked negative assumptions and beliefs adding to fear and cultural misperceptions. Among those most affected are people of Hispanic/LatinX ethnicity, who comprise approximately 18% of the population. To address negative stereotypes, we sought to test how framing affected attitudes about Hispanic/LatinX immigrants and how people in different generations across the political spectrum respond to framing. As part of the “Latinos and Economic Security (LES),” a national research project funded by the Ford Foundation, we launched the “Well Being 501 Latino Economic Security” survey through the American Life Panel of RAND Corporation in 2018. Before answering the survey questions, participants (n=739) were randomly assigned to three different conditions: a 100-word priming statement focused on Hispanic/LatinX work ethic/religiosity/patriotism (33.29%), a 100-word priming statement focused on justice/equity/fairness (32.75%), and a control group with no priming statement (33.96%). We used multiple linear regression to examine relationships among demographic variables, age, political affiliation, and priming statements and attitudes toward Hispanic/LatinX immigrants. Key findings include: 1) age, political affiliation, education level, race and ethnicity, and gender explained 47.5% of the variance in attitudes; 2) baby boomers and generation X were significantly less tolerant of Hispanic/LatinX; 3) priming statements played a salient mediating role in neutralizing negative attitudes. By employing a developmental perspective, we proposed six recommendations from the aspects of reframing policy narrative and developing educational programs targeted at improving attitudes toward Hispanic/LatinX immigrants.

